# Nanotechnology and the Developing World

**DOI:** 10.1371/journal.pmed.0020097

**Published:** 2005-05-12

**Authors:** Fabio Salamanca-Buentello, Deepa L Persad, Erin B Court, Douglas K Martin, Abdallah S Daar, Peter A Singer

## Abstract

How nanotechnology can be harnessed to address some of the world's most critical development problems

Nanotechnology can be harnessed to address some of the world's most critical development problems. However, to our knowledge, there has been no systematic prioritization of applications of nanotechnology targeted toward these challenges faced by the 5 billion people living in the developing world.

In this article, we aim to convey three key messages. First, we show that developing countries are already harnessing nanotechnology to address some of their most pressing needs. Second, we identify and rank the ten applications of nanotechnology most likely to benefit developing countries, and demonstrate that these applications can contribute to the attainment of the United Nations Millennium Development Goals (MDGs). Third, we propose a way for the international community to accelerate the use of these top nanotechnologies by less industrialized countries to meet critical sustainable development challenges.

## Developing Countries Innovate in Nanotechnology

**Figure 1:**
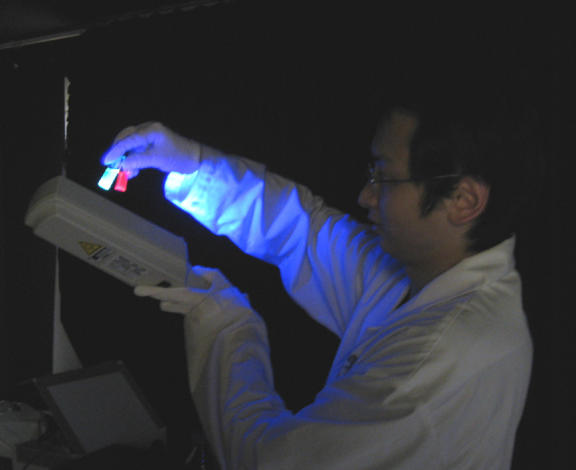
Quantum Dots for Disease Diagnostics Quantum dots may be used for cheap, efficient handheld diagnostic devices available at point-of-care institutions in developing countries.

Several developing countries have launched nanotechnology initiatives in order to strengthen their capacity and sustain economic growth [1]. India's Department of Science and Technology will invest $20 million over the next five years (2004–2009) for their Nanomaterials Science and Technology Initiative [2]. Panacea Biotec (http://www.panacea-biotec.com/products/products.htm) (New Delhi, India) is conducting novel drug delivery research using mucoadhesive nanoparticles, and Dabur Research Foundation (Ghaziabad, India) is participating in Phase-1 clinical trials of nanoparticle delivery of the anti-cancer drug paclitaxel [3]. The number of nanotechnology patent applications from China ranks third in the world behind the United States and Japan [4]. In Brazil, the projected budget for nanoscience during the 2004–2007 period is about $25 million, and three institutes, four networks, and approximately 300 scientists are working in nanotechnology [5]. The South African Nanotechnology Initiative (http://www.sani.org.za) is a national network of academic researchers involved in areas such as nanophase catalysts, nanofiltration, nanowires, nanotubes, and quantum dots (Figure 1). Other developing countries, such as Thailand, the Philippines, Chile, Argentina, and Mexico, are also pursuing nanotechnology [1].

Science and technology alone are not the answer to sustainable development challenges. Like any other science and technology waves, nanoscience and nanotechnology are not “silver bullets” that will magically solve all the problems of developing countries; the social context of these countries must always be considered. Nevertheless, science and technology are a critical component of development [6]. The 2001 Human Development Report [7] of the UN Development Program clearly illustrates the important roles of science and technology in reducing mortality rates and improving life expectancy in the period 1960–1990, but it did not emphasize nanotechnology specifically. In a report released in early 2005 [8], the UN Task Force on Science, Technology and Innovation (part of the process designed to assist UN agencies in achieving the UN MDGs) addresses the potential of nanotechnology for sustainable development.

## Top Ten Nanotechnologies Contributing to the MDGs

In order to provide a systematic approach with which to address sustainable development issues in the developing world, we have identified and ranked the ten applications of nanotechnology most likely to benefit developing countries. We used a modified Delphi Method, as described in our Top Ten Biotechnologies report [9] to identify and prioritize the applications and to achieve consensus among the panelists.

We recruited an international panel of 85 experts in nanotechnology who could provide the informed judgments that this study required, of which 63 completed the project (Table S1). We selected the panelists based on contacts identified in our previous study on nanotechnology in developing countries [1]. A conscious effort was made to balance the panel with respect to gender, specialty areas within nanotechnology, and geographic distribution. Of the panelists, 38 (60%) were from developing countries and 25 (40%) from developed countries; 51 panelists (81%) were male and 12 (19%) were female.

We posed the following open-ended question: “Which do you think are the nanotechnologies most likely to benefit developing countries in the areas of water, agriculture, nutrition, health, energy, and the environment in the next 10 years?” These areas were identified in the 2002 UN Johannesburg Summit on Sustainable Development [10]. We asked the panelists to answer this question using the following criteria derived from our previous Top Ten Biotechnologies study.

**Impact.** How much difference will the technology make in improving water, agriculture, nutrition, health, energy, and the environment in developing countries?

**Burden.** Will it address the most pressing needs?

**Appropriateness.** Will it be affordable, robust, and adjustable to settings in developing countries, and will it be socially, culturally, and politically acceptable?

**Feasibility.** Can it realistically be developed and deployed in a time frame of ten years?

**Knowledge gap.** Does the technology advance quality of life by creating new knowledge?

**Indirect benefits.** Does it address issues such as capacity building and income generation that have indirect, positive effects on developing countries?

Three Delphi rounds were conducted using e-mail messages, faxes, and phone calls. In the first round, the panelists proposed examples of nanotechnologies in response to our study question. We analyzed and organized their answers according to common themes and generated a list of twenty distinct nanotechnology applications. This list was reviewed for face and content validity by two nanotechnologists external to the panel. In the second Delphi round, the panelists ranked their top ten choices from the 20 applications provided and gave reasons for their choices. To analyze the data, we produced a summative point score for each application, ranked the list, and summarized the panelists' reasons. Then we redistributed the top 13 applications, instead of the top ten, to generate a greater number of choices for increased accuracy in the last round. Thus, the highest score possible for an application was 819 (63 × 13). The final Delphi round was devoted to consolidating consensus by re-ranking the top ten of the 13 choices obtained in the previous round and to gathering concrete examples of each application from the panelists.

Our results, shown in Table 1, were compiled from January to July 2004. They display a high degree of consensus with regard to the top four applications: all of the panelists cited at least one of the top four applications in their personal top four rankings, with the majority citing at least three.

**Table 1:**
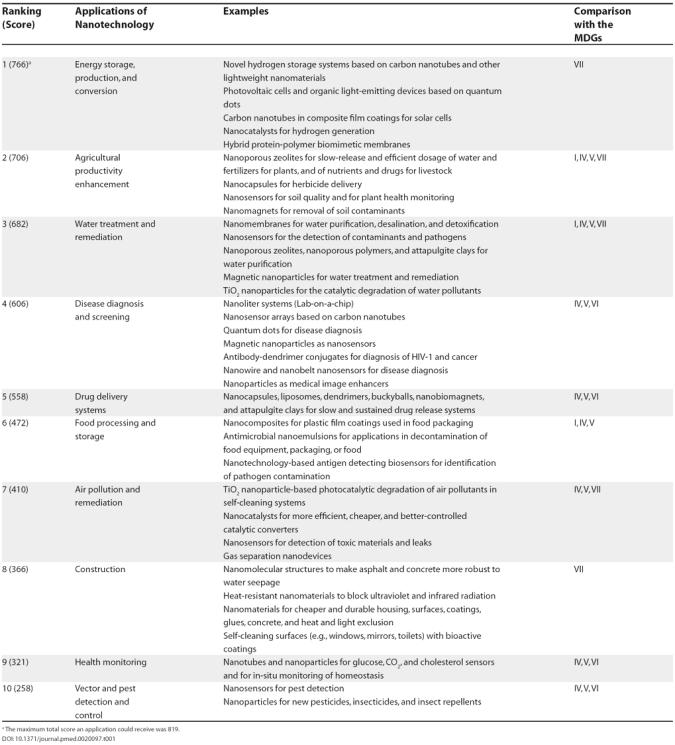
Correlation between the Top Ten Applications of Nanotechnology for Developing Countries and the UN Millennium Development Goals

^a^ The maximum total score an application could receive was 819

**Figure 2:**
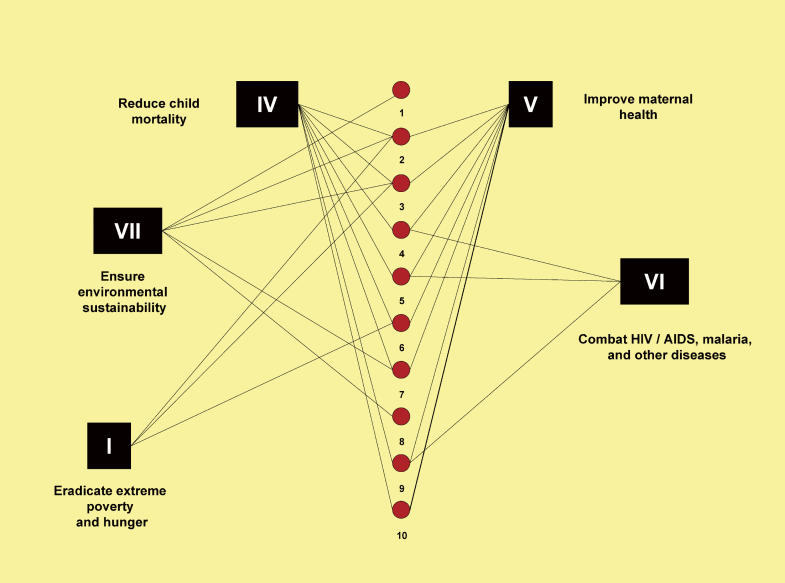
Comparison between the Millennium Development Goals and the Nanotechnologies Most Likely to Benefit Developing Countries in the 2004–2014 Period

To further assess the impact of nanotechnology on sustainable development, we have compared the top ten applications with the UN Millennium Development Goals (Table 1 and Figure 2). The MDGs are eight goals that aim to promote human development and encourage social and economic sustainability [11]. In 2000, all 189 member states of the UN committed to achieve the MDGs by 2015. The MDGs are: (i) Eradicate extreme poverty and hunger; (ii) Achieve universal primary education; (iii) Promote gender equality and empower women; (iv) Reduce child mortality; (v) Improve maternal health; (vi) Combat HIV/AIDS, malaria, and other diseases; (vii) Ensure environmental sustainability; and (viii) Develop a global partnership for development. As shown in Table 1 and Figure 2, the top ten nanotechnology applications can contribute to achieving the UN MDGs.

## Addressing Global Challenges Using Nanotechnology

What can the international community do to support the application of nanotechnology in developing countries? In 2002, the National Institutes of Health (NIH) conceptualized a roadmap for medical research to identify major opportunities and gaps in biomedical investigations. Nanomedicine is one of the areas of implementation that has been outlined to address this concern. Several of the applications of nanotechnology that we have identified in our study can aid the NIH in this process by targeting the areas of research that need to be addressed in order to combat some of the serious medical issues facing the developing world.

To expand on this idea, we propose an initiative, called “Addressing Global Challenges Using Nanotechnology,” to accelerate the use of nanotechnology to address critical sustainable development challenges. We model this proposal on the Foundation for the NIH/Bill and Melinda Gates Foundation's Grand Challenges in Global Health [12], which itself was based on Hilbert's Grand Challenges in Mathematics.

A grand challenge is meant to direct investigators to seek a specific scientific or technological breakthrough that would overcome one or more bottlenecks in an imagined path to solving a significant development problem (or preferably, several) [12]. A scientific board similar to the one created for the Grand Challenges in Global Health, with strong representation of developing countries, will need to be established to provide guidance and oversee the program. The top ten nanotechnology applications identified in Table 1 are a good starting point for defining the grand challenges.

The funding to address global challenges using nanotechnology could come from various sources, including national and international foundations, and from collaboration among nanotechnology initiatives in industrialized and developing countries. These funds could be significantly increased if industrialized nations adopted the target set in February 2004 by Paul Martin, Prime Minister of Canada: that 5% of Canada's research and development investment be used to address developing world challenges [13]. In parallel to the allocation of public funds, policies should provide incentives for the private sector to direct a portion of their research and development toward funding our initiative. The UN Commission on Private Sector and Development report *Unleashing Entrepreneurship: Making Business Work for the Poor* [14] underscores the importance of partnerships with the private sector, especially the domestic private sectors in developing countries, in working to achieve the MDGs.

Perhaps most importantly, our results can provide guidance to the developing countries themselves to help target their growing initiatives in nanotechnology [15]. The goal is to use nanotechnology responsibly [16] to generate real benefits for the 5 billion people in the developing world.

## Supporting Information

Table S1 List of Panel Members (43 KB DOC).

**A Definition of Nanotechnology**

Nanotechnology is the study, design, creation, synthesis, manipulation, and application of functional materials, devices, and systems through control of matter at the nanometer scale (1–100 nanometers, one nanometer being equal to 1 × 10^−9^ of a meter), that is, at the atomic and molecular levels, and the exploitation of novel phenomena and properties of matter at that scale.

## Abbreviations

NIH: National Institutes of Health
MDGs: Millennium Development Goals
