# Supplementary material for: Nanotechnology and the Developing World
Source: PLoS Med. 2005 May 12;2(5):e97. doi: 10.1371/journal.pmed.0020097 (PMC1079975; doi:10.1371/journal.pmed.0020097)
Supplement: Supplementary file 1 [file pmed.0020097.st001.doc]

Table S1. List of Panel Members

| Name | Institutional Affiliation |
| --- | --- |
| Pavadee Aungkavattana | National Metals and Materials Technology Center (Bangkok, Thailand) |
| Angela Belcher | Massachusetts Institute of Technology (Cambridge, Massachusetts, United States) |
| Lalit Bharadwaj | Central Scientific Instruments Organisation (Chandigarh, India) |
| Jyoti S. A. Bhat | Department of Scientific and Industrial Research, Government of India (New Delhi, India) |
| Richard Blaikie | University of Canterbury (Canterbury, New Zealand) |
| Werner J. Blau | Sami Nasr Institute for Advanced Materials (Dublin, Ireland) |
| Jürgen Brugger | École Polytechnique Fédérale de Lausanne (Lausanne, Switzerland) |
| Alejandro Cabrera | Pontificia Universidad Católica de Chile (Santiago, Chile) |
| Myung-Ae Chung | Electronics and Telecommunications Research Institute (Daejeon, South Korea) |
| Fabian M. Dayrit | Ateneo de Manila University (Manila, Philippines) |
| Erwin P. Enriquez | Ateneo de Manila University (Manila, Philippines) |
| Amir A. Farajian | Institute for Materials Research, Tohoku University (Sendai, Japan) |
| Harald Fuchs | Universitæt Muenster (Muenster, Germany) |
| Peter Grütter | McGill University (Montreal, Canada) |
| Albena Ivanisevic | Purdue University (West Lafayette, Indiana, United States) |
| Teerakiat Kerdcharoen | Mahidol University (Nakhon Pathom, Thailand) |
| Phan Hong Khoi | Hanoi National University (Hanoi, Vietnam) |
| Horst Klump | University of Cape Town (Cape Town, South Africa) |
| Gabriel Kolawole | University of Zululand (KwaZulu-Natal, South Africa) |
| SangHo Lim | Korea Institute of Science and Technology (Seoul, South Korea) |
| Dan Maydan | Applied Materials, Inc.; Israel Nanotechnology Trust (Tel Aviv, Israel) |
| Michael Mehta | University of Saskatchewan (Saskatoon, Canada) |
| Paolo Milani | University of Milano (Milano, Italy) |
| Chad Mirkin | Northwestern University (Evanston, Illinois, United States) |
| Carlo Montemagno | University of California, Los Angeles (Los Angeles, California, United States) |
| Mark Morrison | Institute of Nanotechnology (Stirling, United Kingdom) |
| H. S. Nalwa | *Journal of Nanoscience and Nanotechnology* (Stevenson Ranch, California, United States) |
| Androula Nassiopoulou | National Center for Scientific Research (Athens, Greece) |
| Mônica Cristina de Oliveira | Universidade Federal de Minas Gerais (Belo Horizonte, Brasil) |
| Richard Palmer | The University of Birmingham (Birmingham, United Kingdom) |
| Somsak Panyakeow | Chulalongkorn University (Bangkok, Thailand) |
| Christine Peterson | Foresight Institute (Palo Alto, California, United States) |
| Gloria Platero | Consejo Superior de Investigaciones Científicas (Madrid, Spain) |
| Nick Pornsin-Sirirak | National Nanotechnology Center (Thailand Science Park, Thailand) |
| Philippe Poulin | Centre National de la Recherche Scientifique (Paris, France) |
| C. N. R. Rao | Third World Academy of Sciences; Jawaharlal Nehru Centre for Advanced Scientific Research (Jakkur, India) |
| Angel Rubio | Donostia International Physics Center (Donostia-San Sebastian, Spain) |
| Neerish Revaprasadu | University of Zululand (KwaZulu-Natal, South Africa) |
| Caesar Saloma | University of the Philippines (Quezon City, Philippines) |
| Arnel A. Salvador | University of the Philippines (Quezon City, Philippines) |
| Ron Sanderson | University of Stellenbosch (Stellenbosch, South Africa) |
| José Manuel Saniger Blesa | Centro de Ciencias Aplicadas y Desarrollo Tecnológico, UNAM (Mexico City, México) |
| Roland V. Sarmago | University of the Philippines (Quezon City, Philippines) |
| Murali Sastry | National Chemical Laboratory (Pune, India) |
| Ottilia Saxl | Institute of Nanotechnology (Stirling, United Kingdom) |
| Manfred Scriba* | South African Nanotechnology Initiative (Pretoria, South Africa) |
| Swaminathan Sethuraman | SASTRA Deemed University (Thirumalaisamudram, India) |
| Cylon Gonçalves da Silva | Laboratório Nacional de Luz Síncrotron (Campinas, Brasil) |
| Winston Soboyejo | Princeton University (Princeton, New Jersey, United States) |
| Deepak Srivastava | NASA Ames Research Center (Moffett Field, California, United States) |
| Ben Zhong Tang | Hong Kong University of Science and Technology (Hong Kong, China) |
| Wiwut Tanthapanichakoon | Chulalongkorn University (Bangkok, Thailand) |
| Humberto Terrones | Instituto Potosino de Investigación Científica y Tecnológica (San Luis Potosí, México) |
| Alfonso Torres Jácome | Instituto Nacional de Astrofísica, Óptica y Electrónica (Puebla, México) |
| Joaquín Tutor Sánchez | Universidad de La Habana (Havana, Cuba) |
| Pham Hung Viet | Vietnam National University (Hanoi, Vietnam) |
| M. Vijayalakshmi | SASTRA Deemed University (Thirumalaisamudram, India) |
| Antonio Villaflor | Intel (Manila, Philippines) |
| H. Q. Wang | University of Science and Technology of China (Hefei, China) |
| Mitiko Yamaura | Instituto de Pesquisas Energéticas e Nucleares (Sao Paolo, Brasil) |
| Hiroshi Yokoyama | National Institute of Advanced Industrial Science and Technology (Tokyo, Japan) |
| Kyung-Hwa Yoo | Yonsei University (Seoul, South Korea) |
| Christiane Ziegler | University of Kaiserslautern (Kaiserslautern Germany) |

*Manfred Scriba worked in close collaboration with several members of the South African Nanotechnology Initiative.
